# Supplementary material for: Artificial Intelligence in Medical Writing: Subtle Errors and Their Complex Consequences
Source: JMA J. 2026 May 8;9(4):992–6. doi: 10.31662/jmaj.2026-0030 (PMC13453652; doi:10.31662/jmaj.2026-0030)
Supplement: Supplementary file 1 [file 2433-3298-9-4-0992-s001.pdf]

Supplementary File 1

ChatGPT-generated min-review

Placenta Percreta Surgery: From Mortal Threat to Manageable Condition

Placenta accreta spectrum (PAS) disorders encompass a range of pathological placental attachments distinguished by the depth of placental adherence to, and invasion into, the uterine wall. The three classic subtypes are placenta accreta, placenta increta, and placenta percreta, a classification internationally standardised under FIGO criteria [1].

Among these, placenta percreta—defined by villous penetration through the uterine serosa—is the most severe and technically demanding form, carrying the greatest risk of catastrophic haemorrhage, multi-organ injury, and maternal mortality [2]. Therefore requiring meticulous multidisciplinary surgical planning.

Earlier paradigms framed placenta percreta as a fundamentally invasive, cancer-like trophoblastic disease. Contemporary pathophysiological evidence, however, supports a different concept: PAS disorders arise largely from defective uterine remodelling and impaired decidualisation, typically at sites of previous uterine scarring such as caesarean delivery scars, rather than intrinsically aggressive trophoblast behaviour [3]. This conceptual shift—from trophoblastic invasion to uterine abnormality—has influenced surgical management.

Surgical management of placenta percreta has evolved substantially. Caesarean hysterectomy was long considered the default approach because of uncontrollable bleeding and operative risk [4]. More recently, vascular control techniques, including prophylactic aortic endovascular balloon occlusion and resuscitative endovascular balloon occlusion of the aorta (REBOA), have been introduced and shown to reduce intraoperative blood loss in selected PAS cases [5]. Transient aortic clamping and internal iliac artery balloon occlusion or embolisation improved intraoperative haemodynamic stability [6].

Adjunctive haemostatic techniques, including uterine compression sutures, may further reduce perioperative bleeding and facilitate uterine conservation in selected cases [7]. Combining vascular control with focused suturing has been reported to decrease blood loss and transfusion requirements, potentially avoiding hysterectomy [6].

In parallel, uterus-preserving surgical approaches are gaining traction. Segmental uterine resection with reconstruction has been described as a conservative strategy that excises the invaded uterine segment and repairs the defect, with favourable

outcomes reported in carefully selected patients [8]. Placenta-left-in-situ strategies may also reduce immediate surgical morbidity while preserving the uterus, although careful postoperative surveillance remains essential [9].

In conclusion, although placenta percreta was once regarded as a near-insurmountable surgical challenge with high maternal mortality, improved understanding of its pathogenesis, refinement of vascular and haemostatic techniques, and development of conservative surgical options have together transformed surgical outcomes. While centres of excellence remain invaluable, well-prepared secondary-level facilities with appropriate planning and surgical readiness may now achieve favourable outcomes.

References

1. Jauniaux E, Ayres-de-Campos D, Langhoff-Roos J, Fox KA, Collins S; FIGO Placenta Accreta Diagnosis and Management Expert Consensus Panel. FIGO classification for the clinical diagnosis of placenta accreta spectrum disorders. *Int J Gynaecol Obstet.* 2019;146:20-24. PMID: 31173360
2. Hecht JL, Baergen R, Ernst LM, et al. Classification and reporting guidelines for the pathology diagnosis of placentaaccreta spectrum (PAS) disorders: recommendations from an expert panel. *Mod Pathol.* 2020;33(12):2382-2396. PMID: 32415266
3. Jauniaux E, Aplin JD, Fox KA, et al. Placenta accreta spectrum. *Nat Rev Dis Primers.* 2025;11(1):40. PMID: 40473652
4. ACOG. Obstetric Care Consensus No. 7: Placenta accreta spectrum. *Obstet Gynecol*. 2018;132:e259-e275. PMID: 30461695
5. Kluck SL, Russo RM, Appel NB, et al. Aortic balloon occlusion in distal zone 3 reduces blood loss from obstetric hemorrhage in placenta accreta spectrum. *J Trauma Acute Care Surg.* 2023;94(5):710-717. PMID: 36812423
6. D’Antonio F, Iacovelli A, Liberati M, et al. Role of interventional radiology in pregnancy complicated by placenta accreta spectrum disorder: systematic review and meta-analysis. *Ultrasound Obstet Gynecol*. 2019;53:743-751. PMID: 30255598
7. Shih JC, Liu KL, Kang J, Yang JH, Lin MW, Yu CU. 'Nausicaa' compression suture: a simple and effective alternative to hysterectomy in placenta accreta spectrum and other causes of severe postpartum haemorrhage. *BJOG*. 2019;126:412-417. PMID: 30009547

8. Palacios-Jaraquemada JM, Fiorillo A, Hamer J, Martínez M, Bruno C. Placenta accreta spectrum: a hysterectomy can be prevented in almost 80% of cases using a resective-reconstructive technique. *J Matern Fetal Neonatal Med*. 2022;35:275-282. PMID: 31984808
9. Kutuk MS, Ak M, Ozgun MT. Leaving the placenta in situ versus conservative and radical surgery in the treatment of placenta accreta spectrum disorders. *Int J Gynaecol Obstet.* 2018;140:338-344. PMID: 28833092

Supplementary File 2

Experiment on “hyperlipidemia”

Prompt

I am preparing a short educational article for early-career generalists on hyperlipidaemia/hypercholesterolaemia. Please draft a manuscript of approximately 300 words.

1. Please incorporate the bullet points that I will provide.
2. The conclusion should emphasise that hyperlipidaemia/hypercholesterolaemia is a common condition and that long-term follow-up within a sustained doctor–patient relationship is essential.
3. You may include additional points that you consider relevant and helpful for this audience.

Bullet points

1. Hyperlipidaemia is a common condition in the general population and is an important risk factor for cardiovascular and cerebrovascular ischaemic events.
2. Owing in part to westernised dietary habits and lifestyle changes, the prevalence of hyperlipidaemia has been increasing in Japan.
3. Hyperlipidaemia is frequently detected during routine medical check-ups. Japanese guidelines recommend management strategies that include dietary modification, physical activity, and pharmacological therapy, with treatment intensity determined by overall cardiovascular risk. In clinical practice, many physicians initially recommend lifestyle modification and monitor the response over time.

4. Given that statins are generally safe but not always immediately indicated in low- to moderate-risk patients, and considering the need for cost-effective healthcare delivery in Japan, a stepwise approach beginning with lifestyle intervention and follow-up is often appropriate.
5. Because hyperlipidaemia is typically a long-term and often asymptomatic condition, sustained follow-up is necessary, and a stable doctor–patient relationship plays a particularly important role.
6. Beyond the bullet points provided, please include any mandatory issues (especially clinically important issues) that general practitioners should be aware of when they encounter patients with hyperlipidaemia or hypercholesterolaemia.

ChatGPT-generated manuscript

Hyperlipidaemia in General Practice: Practical Considerations for Early-Career Physicians

Hyperlipidaemia is a common condition in the general population and a well-established risk factor for cardiovascular and cerebrovascular ischaemic events. Because of its high prevalence and largely silent course, it is frequently encountered in general practice and represents a core area of long-term preventive care.

In Japan, the prevalence of hyperlipidaemia has increased over recent decades. Westernised dietary habits, reduced physical activity, and broader lifestyle changes are thought to contribute to this trend. As a result, abnormal lipid profiles are often detected during routine medical check-ups, sometimes in patients who feel entirely well and have no specific complaints.

Japanese guidelines recommend a comprehensive management strategy that includes dietary modification, regular physical activity, and pharmacological therapy when indicated. Importantly, treatment intensity is determined by the patient's overall cardiovascular risk rather than lipid levels alone. In everyday clinical practice, many physicians initially recommend lifestyle modification and monitor the response over time, particularly in patients at low to moderate risk.

Statins are generally safe and highly effective, but they are not always immediately required. In addition to clinical considerations, cost-effective healthcare delivery is an important issue in Japan. For this reason, a stepwise approach—beginning with lifestyle intervention, followed by careful follow-up and reassessment—is often appropriate.

General practitioners should remain attentive to secondary causes of dyslipidaemia, adherence to lifestyle advice, and the potential for drug–drug interactions once pharmacotherapy is introduced.

Hyperlipidaemia is typically a long-term and often asymptomatic condition. Short-term improvement in laboratory values should not be mistaken for resolution. Sustained follow-up is therefore essential, not only to monitor lipid levels but also to reinforce lifestyle measures and reassess cardiovascular risk as patients age or develop comorbidities.

In conclusion, hyperlipidaemia and hypercholesterolaemia are common conditions that require patience rather than urgency, and continuity rather than episodic care. A stable, long-term doctor–patient relationship provides the foundation for effective follow-up, shared decision-making, and meaningful risk reduction over time.
